# Differential Protein Expression in Congenital and Acquired Cholesteatomas

**DOI:** 10.1371/journal.pone.0137011

**Published:** 2015-09-03

**Authors:** Seung-Ho Shin, Mei Huang, Sung Huhn Kim, Jae Young Choi

**Affiliations:** 1 Department of Otorhinolaryngology–Head and Neck Surgery, School of Medicine, Ewha Womans University, Seoul, Korea; 2 Department of Medicine, Graduate School, Yonsei University, Seoul, Korea; 3 Research Center for Human Natural Defense System, Yonsei University College of Medicine, Seoul, Korea; 4 Department of Otorhinolaryngology, Yonsei University College of Medicine, Seoul, Korea; University of Alabama at Birmingham, UNITED STATES

## Abstract

Congenital cholesteatomas are epithelial lesions that present as an epithelial pearl behind an intact eardrum. Congenital and acquired cholesteatomas progress quite differently from each other and progress patterns can provide clues about the unique origin and pathogenesis of the abnormality. However, the exact pathogenic mechanisms by which cholesteatomas develop remain unknown. In this study, key proteins that directly affect cholesteatoma pathogenesis are investigated with proteomics and immunohistochemistry. Congenital cholesteatoma matrices and retroauricular skin were harvested during surgery in 4 patients diagnosed with a congenital cholesteatoma. Tissue was also harvested from the retraction pocket in an additional 2 patients during middle ear surgery. We performed 2-dimensional (2D) electrophoresis to detect and analyze spots that are expressed only in congenital cholesteatoma and matrix-assisted laser desorption/ionization time of flight mass spectrometry (MALDI-TOF/MS) to separate proteins by molecular weight. Protein expression was confirmed by immunohistochemical staining. The image analysis of 2D electrophoresis showed that 4 congenital cholesteatoma samples had very similar protein expression patterns and that 127 spots were exclusively expressed in congenital cholesteatomas. Of these 127 spots, 10 major spots revealed the presence of titin, forkhead transcription activator homolog (FKH 5–3), plectin 1, keratin 10, and leucine zipper protein 5 by MALDI-TOF/MS analysis. Immunohistochemical staining showed that FKH 5–3 and titin were expressed in congenital cholesteatoma matrices, but not in acquired cholesteatomas. Our study shows that protein expression patterns are completely different in congenital cholesteatomas, acquired cholesteatomas, and skin. Moreover, non-epithelial proteins, including FKH 5–3 and titin, were unexpectedly expressed in congenital cholesteatoma tissue. Our data indicates that congenital cholesteatoma origins may differ from those of acquired cholesteatomas, which originate from retraction pocket epithelia.

## Introduction

A congenital cholesteatoma is a keratinized squamous epithelial middle ear lesion that usually presents in young children who do not have a history of otitis media, middle ear surgery, or trauma. The presentation and progress of congenital cholesteatoma are quite different from those of acquired cholesteatoma. Congenital cholesteatomas commonly appear as pearl-like epidermoid cysts, mostly located in the antero-superior quadrant behind an intact tympanic membrane [1, 2]. These cholesteatomas can have invasive growth and cause osteolysis [3].

The pathogenesis of congenital cholesteatomas seems to be different from acquired cholesteatomas, which usually arise from the retraction pocket of the pars flaccida of tympanic membrane and have attic destruction [4, 5]. Several pathogenic mechanisms of congenital cholesteatoma have been suggested, including epithelial rests from faulty embryogenesis, invagination or implantation of squamous epithelium, and metaplasia of middle ear epithelium [6]. These suggested pathogenic mechanisms are based on temporal bone histology studies [7–12]. There have been several studies that investigated differences in molecular properties between congenital and acquired cholesteatomas. Upregulation of P21, shorter telomere length, increased surviving expression, and absence of ICAM-1 expression and LFA-1 positive cells have all been suggested to play a role in congenital cholesteatoma pathogenesis. Unfortunately, their exact roles have not been fully identified [3–5, 13]. These studies investigated several target proteins, but no reports have identified differences in molecular expression between congenital and acquired cholesteatoma by mass screening of proteins in each tissue, largely because of the lack of an ideal animal model and a limited number of specimens, which restricted basic molecular and biochemical research on congenital cholesteatoma pathogenesis.

Here, we investigate differences in protein expression between congenital and acquired cholesteatomas using proteomic analysis (for protein mass screening) and immunohistochemistry. Our data suggest that protein expression patterns in congenital cholesteatomas are quite different from those in acquired cholesteatomas and skin. The results of our study may provide clues that allow for a better understanding of congenital cholesteatoma pathogenesis.

## Materials and Methods

### Tissue harvest

**Fig 1:**
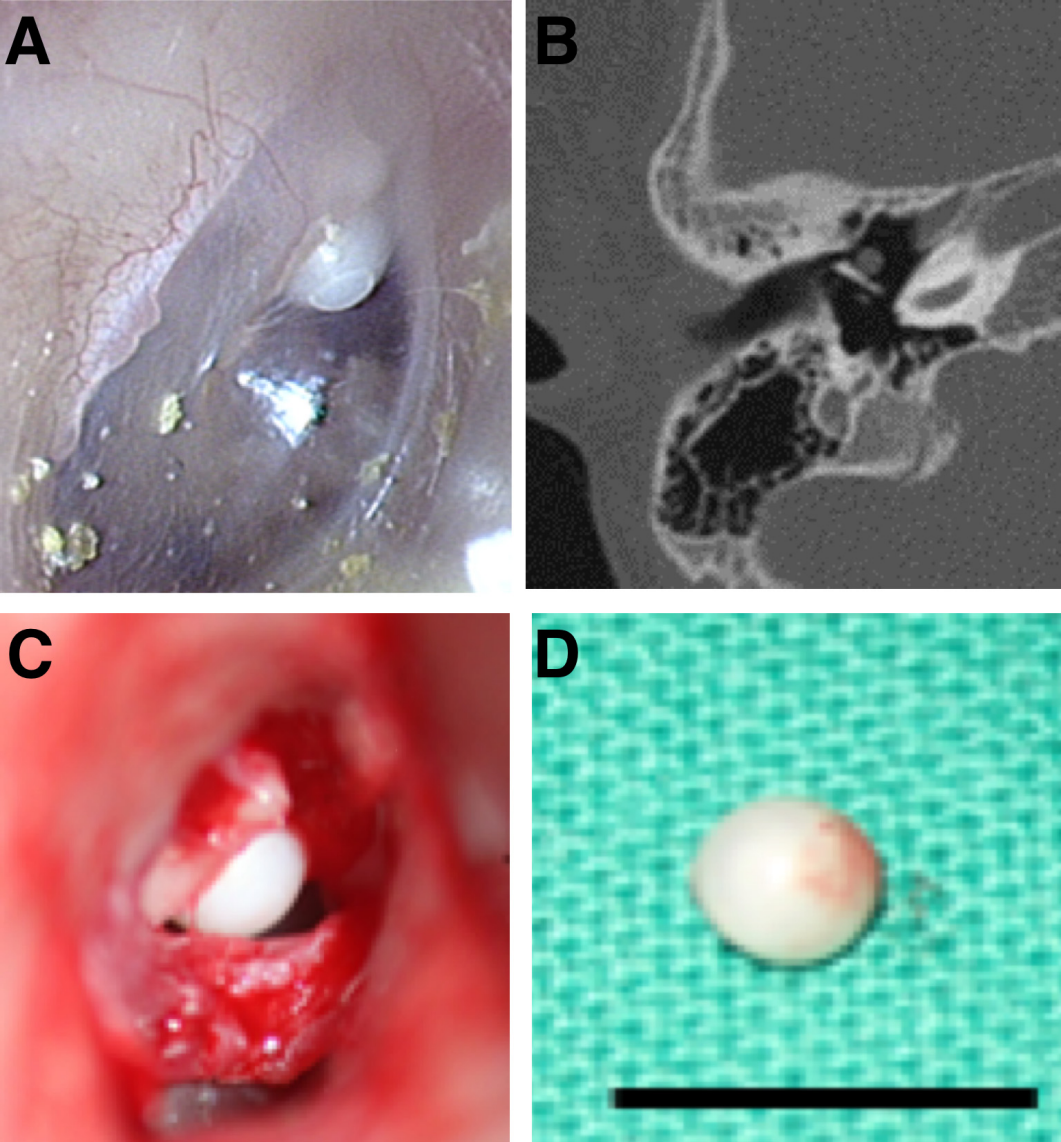
A representative case of congenital cholesteatomas examined in this study (Patient 2 in Table 1). A. Otoscopic tympanic membrane findings. B. Axial computed tomography image of the temporal bone. C. Surgical findings. D. Congenital cholesteatoma specimen obtained in surgery (scale bar: 5mm).

Patients were enrolled in this study after they or their parents provided written informed consent. This study was approved by the institutional review board of Severance Hospital. Samples of congenital cholesteatoma and retroauricular skin were harvested from 6 male children between 4 and 5 year old. All congenial cholesteatomas presented as spherical epithelial pearls behind an intact eardrum in the anterior superior quadrant (Fig 1). In all cases, the congenital cholesteatoma was removed from the middle ear cavity without damaging its capsule. The tympanomeatal flap was elevated using the retroauricular approach. A small amount of retroauricular skin (5 mm x 3 mm ellipsoid) was harvested as a control sample. Samples of acquired cholesteatomas in the pars flaccida area of tympanic membrane were also harvested from 2 adult patients during tympanomastoid surgery (Table 1).

**Table 1:** Study subjects and specimen descriptions.

Subject No.	Gender/age	Specimen type	Specimen description
1	Male/4 years	CC and RAS	3.0 mm diameter epithelial pearl
2	Male/4 years	CC and RAS	2.5 mm diameter epithelial pearl
3	Male/4 years	CC and RAS	3.5 mm diameter epithelial pearl
4	Male/5 years	CC and RAS	3.0 mm diameter epithelial pearl
5	Male/5 years	CC and RAS	3.5 mm diameter epithelial pearl
6	Male/5 years	CC and RAS	3.0 mm diameter epithelial pearl
5	Female/29 years	AC	2 x 2 mm retraction pocket
6	Female /51 years	AC	2 x 3 mm retraction pocket

AC, acquired cholesteatoma; CC, congenital cholesteatoma; RAS, retroauricular skin.

Four harvested samples (subject 1–4 in Table 1) were placed in a small tube, immediately frozen in liquid nitrogen, and stored at-80°C until the time of experiment for proteomic analysis. Two samples for immunohistochemistry (subject 5 and 6 in Table 1) studies were stored in 4% paraformaldehyde. All samples were gently washed with normal saline 3–4 times to remove contaminated tissue and blood.

### Sample preparation for 2-Dimensional Electrophoresis

Samples for 2-dimensional electophoresis (2-DE) were suspended in 4x sample buffer (7 M urea, 2 M thiourea, 4% CHAPS, 0.5% ampholyte, 100 mM dithiothretol, 40 mM Tris, 0.002% bromophenol blue) and sonicated. Then, 10μM DNase solution was added and the sample was incubated for 30 minutes at 4°C. After incubation, samples were centrifuged (105,000 G) for 45 minutes. Next, 50% trichloroacetic acid (TCA; Sigma, St. Louis, MO, USA) was added to obtain a final TCA concentration of 5–8%. Samples were then incubated for 2 hours on ice. After incubation, 200 μL of cold acetone was added and the protein pellet was resuspended with a pipette. Samples were again incubated on ice for 15 minutes and centrifuged (14,000 G) for 20 minutes, after which the acetone was discarded. Finally, the protein pellet was air-dried and dissolved in 200 μL sample buffer for quantification with Bradford methods.

### Two-Dimensional Electrophoresis

All 2-DE was performed as described [14]. Briefly, aliquots in sample buffer (260 μg) were applied to immobilized pH 3–10 nonlinear gradient strips (Amersham Biosciences, Uppsala, Sweden). Isoelectric focusing was performed at 80,000 Vh. The second dimension was analyzed on 9–16% linear gradient polyacrylamide gels (18 cm × 20 cm × 1.5 cm) at a constant 40 mA per gel for 5 hours. After protein fixation in 40% methanol and 5% phosphoric acid for 1 hour, gels were stained with Coomassie Brilliant Blue G-250 for 12 hours. Gels were then destained with H_2_O, scanned in a Bio-Rad GS710 densitometer (Bio-Rad, Hercules, CA, USA), and converted into electronic files (12 bit tiff).

### 2-DE image analysis

Detection of individual spots and measurement of their volume (%) was performed with Image Master Platinum 5 software (GE Healthcare, Piscataway, NJ). Protein spots exclusively identified in congenital cholesteatoma samples were analyzed.

### Protein Identification with Mass Spectrometry

Among the exclusive spots identified by 2-DE image analysis, protein components of the 10 most prominent spots were investigated using matrix-assisted laser desorption/ionization time of flight mass spectrometry (MALDI-TOF MS). For 2-DE gel mapping, major proteins were identified by mass finger printing. Protein spots excised from 2-DE gels were destained, reduced, alkylated, and digested with trypsin (Promega, Madison, WI), as previously described [15]. For MALDI-TOF MS analyses, peptides were concentrated with a POROS R2, Oligo R3 column (Applied Biosystems, Foster city, CA, USA). After washing the column with 70% acetonitrile, 100% acetonitrile, and 50 mM ammonium bicarbonate, samples were applied to the R2, R3 column and eluted onto the MALDI plate (Opti-TOF 384-well Insert, Applied Biosystems) with cyano-4-hydroxycinnamic acid (CHCA; Sigma, St. Louis, MO) dissolved in 70% acetonitrile and 2% formic acid [16]. MALDI-TOF MS was performed on 4800 MALDI-TOF/TOF Analyzer (Applied Biosystems) equipped with a 355 nm Nd:YAG laser. The pressure in the TOF analyzer was approximately 7.6 x 10^–7^ Torr. Mass spectra were obtained in the reflectron mode with an accelerating voltage of 20 kV, summed from 500 laser pulses, and calibrated using the 4700 calibration mixture (Applied Biosystems). Proteins were identified from the peptide mass maps using MASCOT (http://www.matrixscience.com/search_form_select.html), which searched the 115,818 entries in the protein databases of the National Center for Biotechnology Information (NCBI) non-redundant human database (downloaded on 05/09/2009).

### Immunohistochemical staining

To confirm titin and FKH 5–3 protein expression in congenital cholesteatomas, tissues were fixed with 4% paraformaldehyde for 24 hours, dehydrated, and embedded in paraffin. Paraffin blocks were sectioned into 5 μm thick slices and fixed with a chilled 1:1 mixture of methanol:acetone for 5 minutes after pretreatment with 0.3% H_2_O_2_ for 20 minutes at room temperature. Slides were treated with 1:600 normal rabbit serum for 20 minutes to block nonspecific reactions and then incubated with a polyclonal antibody against target human proteins, including titin (1:200, HPA007042, Sigma, St. Louis, MO, USA) and forkhead transcription activator homolog (1:200, clone FKH 5–3, human (fragment), AHP933, AbD Serotec, Kidlington, England). Slides were then incubated with biotinylated antihuman rabbit immunoglobulin G (1:200; Vector Laboratories, Burlingame, CA, USA). For negative controls, the step in which samples were reacted with primary antibodies (titin and clone FKH 5–3) was skipped. Peroxidase was attached to the secondary antibody by avidin-biotin peroxidase complex formation. Specimens were incubated in diaminobenzidine tetrahydrochloride to detect primary antibody binding sites.

## Results

### Differential protein expression in congenital cholesteatoma, acquired cholesteatoma, and external canal skin

**Fig 2:**
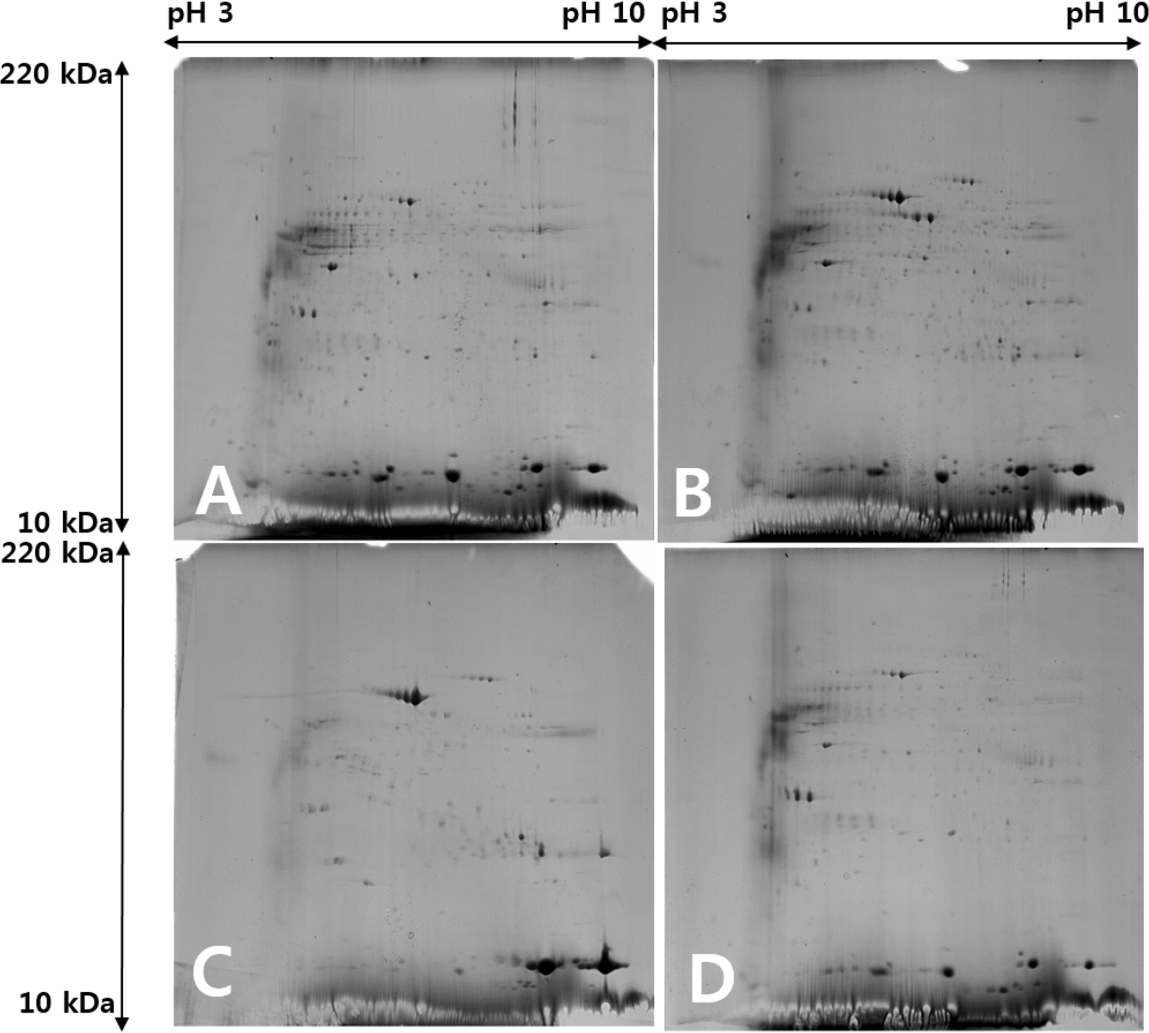
Expression pattern of protein spots identified with 2-dimensional electrophoresis of congenital cholesteatoma. Expression patterns in the 4 patients examined were similar (A-D).

**Fig 3:**
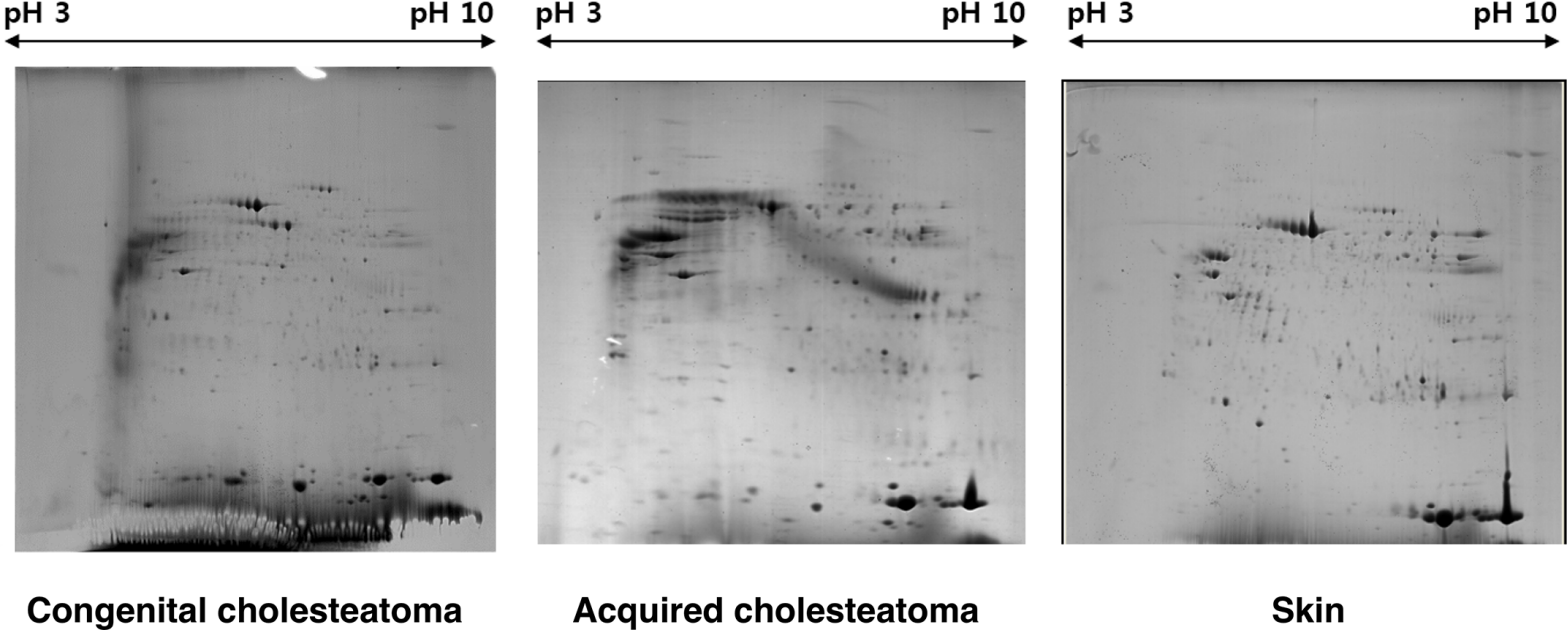
Protein expression patterns in congenital cholesteatoma (left), acquired cholesteatomas (middle), and the retroauricular canal skin (right).

**Fig 4:**
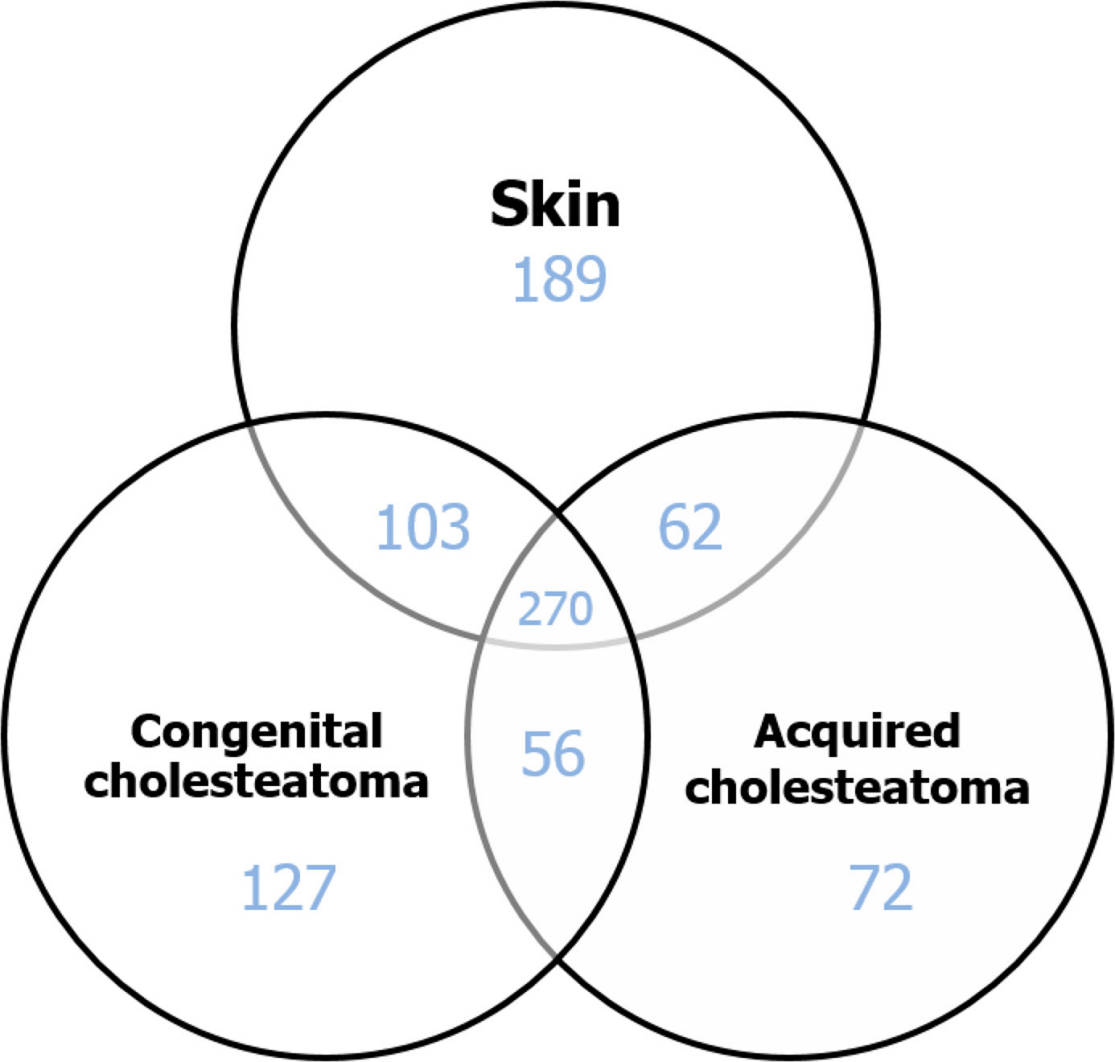
Venn diagram showing the number of identified protein spots in each tissue. One hundred twenty seven spots were only expressed in congenital cholesteatoma.

The 2-DE analysis of the four congenital cholesteatoma samples showed very similar protein expression patterns (Fig 2) that were quite different from acquired cholesteatoma and retroauricular skin samples (Fig 3). A total of 556 spots were identified in congenital cholesteatomas from 2-DE images analysis. Of the 556 spots, 270 were also simultaneously expressed in acquired cholesteatoma and EAC skin. Additionally, 103 and 56 spots were expressed in skin and acquired cholesteatoma, respectively. Finally, 127 spots were only expressed in congenital cholesteatoma (Fig 4).

### Main proteins expression in congenital cholesteatoma

**Fig 5:**
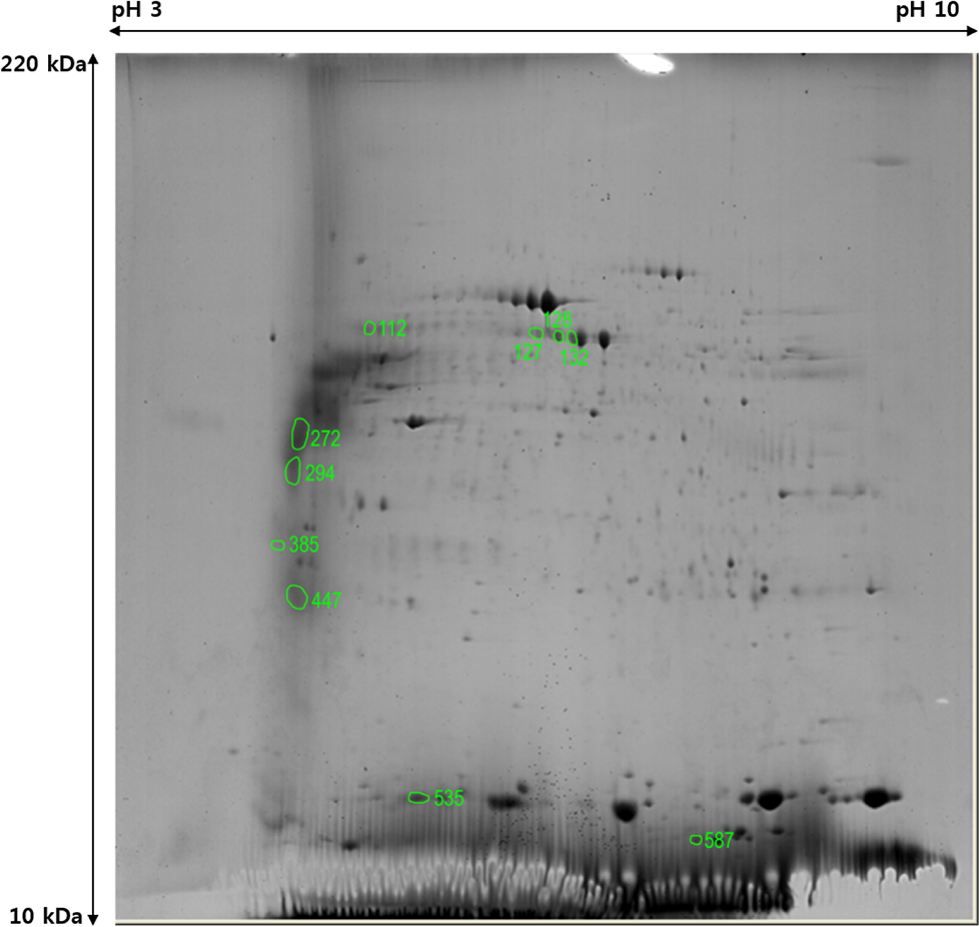
Ten major spots only expressed in congenital cholesteatoma, as determined by image analysis.

The MALDI-TOF MS analyses were performed to identify the 10 most abundant spots (out of 127 spots) that were only expressed in congenital cholesteatoma (Fig 5). These proteins are summarized in Table 2 and included titin (gi|407139), PRO2619 (gi|11493459), forkhead transcription activator homolog (gi|477361, FHK 5–3), ryanodine receptor 2 isoform CRA_c (gi|119590477), plectin 1 intermediate filament binding protein (gi|119602578), keratin 10 (epidermolytic hyperkeratosis; keratosis palmaris et plantaris, gi|119581085), keratin 10 (gi|186629), keratin 10 (gi|119581085), titin (gi|407139), and leucine zipper protein 5 isoform CRA_b (gi|119624991).

**Table 2:** 10 major protein exclusively expressed in congenital cholesteatoma.

Spot No.	gi number	Protein name	M_r_	PI	% coverage	Matched peptide number
112	gi|407139	titin	524823	8.06	10	28
127	gi|11493459	PRO2619	58513	5.96	21	15
128	gi|477361	FKH 5–3	12861	10.06	66	5
132	gi|119590477	ryanodine receptor 2 (cardiac) isoform CRA_c	568496	5.69	10	28
272	gi|119602578	plectin 1 intermediate filament binding protein isoform CRA_c	290791	5.62	14	41
294	gi|119581085	keratin 10	63536	5.13	28	16
385	gi|186629	keratin 10	39832	4.72	31	10
447	gi|119581085	keratin 10	63536	5.13	30	16
535	gi|407139	titin	524823	8.06	11	29
587	gi|119624991	leucine zipper protein 5 isoform CRA_b	9456	11.70	72	8

M_r_, nominal mass; FKH, forkhead transcriptional factor; PI, calculated PI value; %coverage, sequence coverage; matched peptide number, number of mass values matched.

### Forkhead transcription factor (FKH 5–3) and titin expression in congenital cholesteatoma

**Fig 6:**
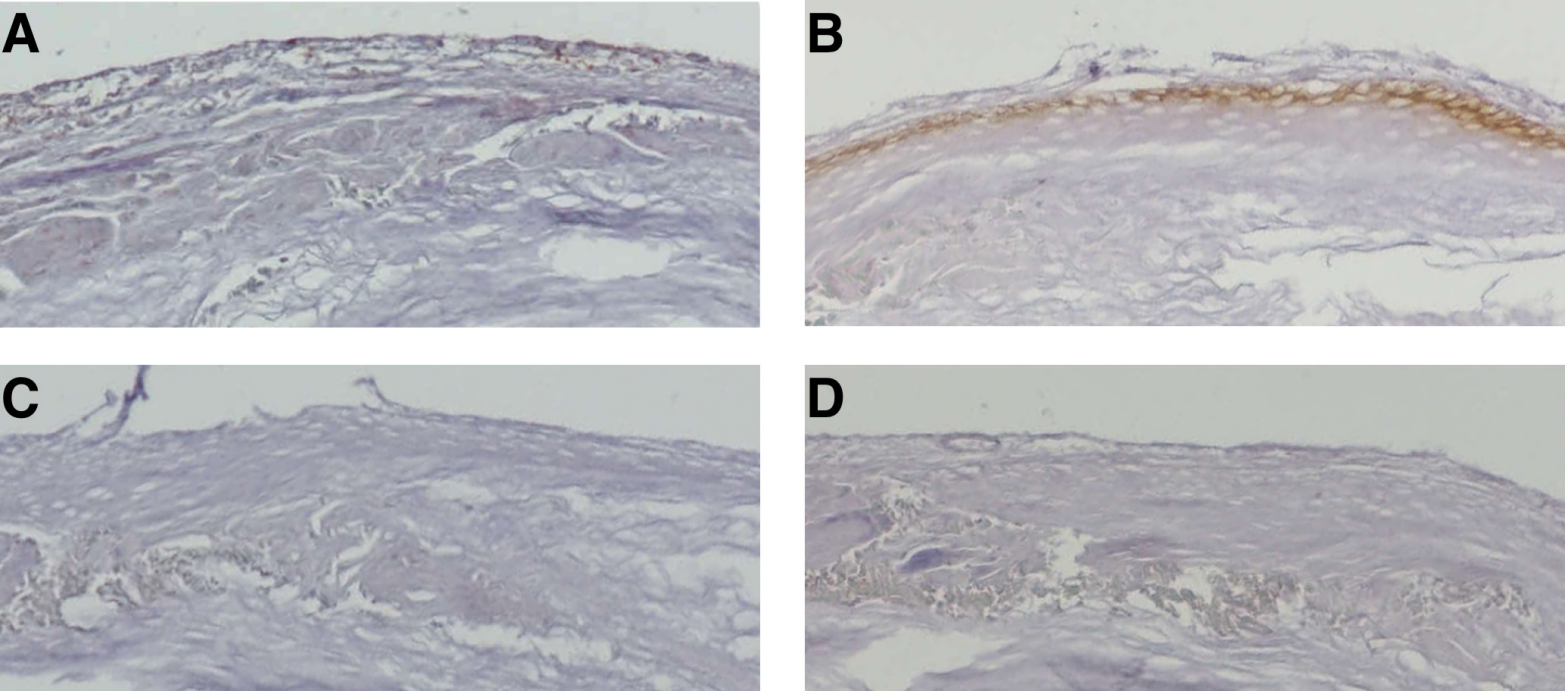
Expression of forkhead transcriptional factor homolog (FKH 5–3, A) and titin (B) in congenital cholesteatoma, as detected with immunohistochemistry. Both proteins were well-expressed in cholesteatoma matrix. Negative controls (C, D) are also shown for comparison.

Among the proteins confirmed by MALDI-TOF MS, FKH 5–3 and titin proteins were selected based on antibody availability and connection probability between previously described pathogenesis and known protein characteristics. Immunolocalization demonstrated that FKH 5–3 and titin were localized in the cell membrane and cytoplasm in all layers of congenital cholesteatoma keratinocytes (Fig 6).

## Discussion

Recent studies of cholesteatoma have focused on acquired cholesteatoma [1, 17, 18]. A relatively small number of studies have been published on congenital cholesteatoma. To the best of our knowledge, no prior studies have used proteomics to investigate congenital cholesteatoma pathogenesis.

Our study showed that protein spot 2-DE distribution patterns of congenital cholesteatoma are consistent among specimens and completely different from acquired cholesteatoma. Many proteins (127 spots) were exclusively expressed in congenital cholesteatoma, and not found in EAC skin or acquired cholesteatoma. Proteins that we did not expect to be expressed in fully differentiated epithelial cells were titin (gi|407139), forkhead transcription activator homolog (FKH 5–3, gi|477361), ryanodine receptor 2 (cardiac), and isoform CRA_c (gi|119590477), all of which are expressed in mesodermal tissues [19–21].

Several theories have been proposed regarding congenital cholesteatoma origin, including the ‘epithelial rest theory’ [10], the ‘invagination theory’ [22, 23], and the ‘implantation theory’ [9]. Although it remains unclear which theory best describes congenital cholesteatoma etiology, all theories insist that congenital and acquired cholesteatoma have different origins. Aquired cholesteatoma generally arise from a pars flaccida retraction pocket.

Unexpected expression of non-epithelial protein in our experiments contradicts the epithelial rest theory where the congenital cholesteatoma arises from ectodermal derivatives from the first epibranchial placode, as found in various vertebrates. Among the 10 most abundant proteins exclusively expressed in congenital cholesteatoma tissues, FKH 5–3 was most interesting. Expression of this protein was also confirmed in congenital cholesteatoma by immunohistochemistry. Forkhead proteins, a family of transcription factors, play pathophysiologic roles in regulating expression of genes involved in cell growth, proliferation, differentiation, and longevity. Forkhead proteins regulate embryonic development mechanisms [24] and are expressed in hematopoietic stem cells that originate from the mesoderm and regulate lymphocyte development [20]. It has also been reported that forkhead transcription factors can be a downstream target of the Akt/PKB pathway, which is activated by microRNA 21 upregulation and subsequent apoptosis inhibition [25]. The presence of FKH 5–3 might indicate abnormal cell growth and be the pathogenic origin of congenital cholesteatoma.

Another distinctive finding of our study was expression of titin in congenital cholesteatoma. Titin is exclusively expressed in muscle [26] and originates from the mesoderm, where it plays a key role in vertebrate striated muscle assembly and function. Recent studies also found that titin is expressed in chromosomes and has functions related to oncogenesis [27–30]. We found that titin matched the major spots found in proteomic analysis on congenital cholesteatoma samples and was also strongly expressed in cholesteatoma matrices, as shown with immunostaining. The role of these proteins remain unclear in pathophysiology of congenital cholesteatoma, but we suggest it may be involved in congenital cholesteatoma cell proliferation.

Although several markers indirectly suggest pathogenic mechanisms of congenital cholesteatoma, our study has two main limitations. First, proteomics studies have inherent limitations. The amount of expression was determined only by image analysis, which could have differed if a more delicate experimental method was used. Therefore, there could be more abundant proteins that exclusively exist in congenital cholesteatoma. In addition, we only investigated the 10 most abundant spots identified in 2-DE analysis. However, there could be more important proteins that play a larger role in congenital cholesteatoma pathogenesis. We were only able to theorize congenital cholesteatoma pathogenic mechanisms using exclusive protein expression found by proteomic analysis. However, no information was gained on the role or importance of identified proteins in pathogenesis, which require animal models and/or in vitro cell culture systems. Second, limitations arose from working with harvested tissue. Congenital cholesteatoma tissue forms a cystic pearl, which was properly washed. Therefore, there should have been little contamination from surrounding tissue. However, sample contamination by middle ear mucosa cannot be completely excluded in acquired cholesteatoma because acquired cholesteatomas are located in retraction pockets and strongly adhere to middle ear mucosa. There also could have been contamination with blood even though surface blood was removed by extensive tissue washing (5–6 times in normal saline until blood no longer visible).

Even with these limitations, our proteomic study on congenital cholesteatoma tissue provides some clues on cholesteatoma pathogenesis. It is tempting to speculate that the origin of congenital cholesteatomas differs from that of acquired cholesteatomas, which originate from tympanic membrane epithelium. This study provides justification for future research on congenital cholesteatoma pathogenesis.
